# Protective Effects of Fucoxanthin on Hydrogen Peroxide-Induced Calcification of Heart Valve Interstitial Cells

**DOI:** 10.3390/md19060307

**Published:** 2021-05-26

**Authors:** Yi-Fen Chiang, Chih-Hung Tsai, Hsin-Yuan Chen, Kai-Lee Wang, Hsin-Yi Chang, Yun-Ju Huang, Yong-Han Hong, Mohamed Ali, Tzong-Ming Shieh, Tsui-Chin Huang, Ching-I Lin, Shih-Min Hsia

**Affiliations:** 1 School of Nutrition and Health Sciences, College of Nutrition, Taipei Medical University, Taipei 11031, Taiwan; yvonne840828@gmail.com (Y.-F.C.); d507104002@tmu.edu.tw (H.-Y.C.); d04641004@ntu.edu.tw (Y.-J.H.); 2 Yu-Kang Animal Hospital, New Taipei City 220, Taiwan; yukangdvm@yahoo.com.tw; 3 Department of Nutrition, I-Shou University, Kaohsiung 84001, Taiwan; yonghan@isu.edu.tw; 4 Department of Nursing, Ching Kuo Institute of Management and Health, Keelung 20301, Taiwan; kellywang@tmu.edu.tw; 5 Graduate Institute of Metabolism and Obesity Sciences, Taipei Medical University, Taipei 11031, Taiwan; hsinyi.chang@tmu.edu.tw; 6 Clinical Pharmacy Department, Faculty of Pharmacy, Ain Shams University, Cairo 11566, Egypt; mohamed.aboouf@pharma.asu.edu.eg; 7 School of Dentistry, College of Dentistry, China Medical University, Taichung 40402, Taiwan; tmshieh@mail.cmu.edu.tw; 8 Graduate Institute of Cancer Biology and Drug Discovery, College of Medical Science and Technology, Taipei Medical University, Taipei 11031, Taiwan; tsuichin@tmu.edu.tw; 9 Department of Nutrition and Health Sciences, Kainan University, Taoyuan 338, Taiwan; cilin@mail.knu.edu.tw; 10 School of Food and Safety, Taipei Medical University, Taipei 11031, Taiwan; 11 Nutrition Research Center, Taipei Medical University Hospital, Taipei 11031, Taiwan

**Keywords:** fucoxanthin, oxidative stress, calcification, heart valve interstitial cell

## Abstract

Cardiovascular diseases such as atherosclerosis and aortic valve sclerosis involve inflammatory reactions triggered by various stimuli, causing increased oxidative stress. This increased oxidative stress causes damage to the heart cells, with subsequent cell apoptosis or calcification. Currently, heart valve damage or heart valve diseases are treated by drugs or surgery. Natural antioxidant products are being investigated in related research, such as fucoxanthin (Fx), which is a marine carotenoid extracted from seaweed, with strong antioxidant, anti-inflammatory, and anti-tumor properties. This study aimed to explore the protective effect of Fx on heart valves under high oxidative stress, as well as the underlying mechanism of action. Rat heart valve interstitial cells under H_2_O_2_-induced oxidative stress were treated with Fx. Fx improved cell survival and reduced oxidative stress-induced DNA damage, which was assessed by cell viability analysis and staining with propidium iodide. Alizarin Red-S analysis indicated that Fx has a protective effect against calcification. Furthermore, Western blotting revealed that Fx abrogates oxidative stress-induced apoptosis via reducing the expression of apoptosis-related proteins as well as modulate Akt/ERK-related protein expression. Notably, in vivo experiments using 26 dogs treated with 60 mg/kg of Fx in combination with medical treatment for 0.5 to 2 years showed significant recovery in their echocardiographic parameters. Collectively, these in vitro and in vivo results highlight the potential of Fx to protect heart valve cells from high oxidative stress-induced damage.

## 1. Introduction

The pathogenesis of cardiovascular disease, such as atherosclerosis and aortic valve sclerosis, involves inflammatory reactions in response to a variety of stimuli including high levels of low-density lipoprotein (LDL) and reactive oxygen species (ROS). The latter are triggered by increased oxidative stress, infections, and chemical damage. In cardiovascular diseases, increased load on myocardial cells or insufficient energy supply would cause an imbalance in energy supply and demand, resulting in an increase in energy metabolism and mitochondrial redox. This eventually leads to an increase in reactive oxygen species (ROS) and damage to heart cells [1].

Heart valves, such as the aortic valve, mainly comprise two types of cells: valve endothelial cells (VEC) on the surface and valve interstitial cells (VIC) in the matrix. VECs regulate message transmission and permeability and also prevent thrombosis [2]. VICs maintain the tissue structure of the valves. Normally, healthy valve cells are mainly of the fibroblast type, but cell apoptosis can be induced in cases of increased reactive oxygen species and this leads to valve damage [3]. Apoptosis is a regulatory pathway of programmed cell death in response to signals generated by environmental stimuli. In the process of apoptosis, a DNA repair protein called poly (ADP-ribose) polymerase-1 (PARP-1) is truncated, causing cell apoptosis [4].

Another role of elevated ROS is in affecting extracellular matrix (ECM) remodeling [5]. In heart valves, ECM remodeling occurs in layers enriched with VIC fibroblast cells with subsequent valve fibrosis potential. A previous study showed that valvular osteoblast induction could activate the ECM accumulation and calcification process [6,7]. Moreover, ROS-induced chronic inflammation resulted in an influx of macrophages and increased pro-osteogenic and angiogenic activities [5], which in return increased the production of proteolytic enzymes and triggered ECM remodeling and valve fibrosis [8].

Small-breed dogs weighing less than 20 kg can suffer from heart valve diseases [9]. In Taiwan, Maltese present the highest incidence [10]. The progression of valve disease is correlated with increased age, heart volume enlargement, and the regurgitation of blood flow [10].

Fucoxanthin (Fx) is a carotenoid with high anti-oxidative activity that is abundant in brown seaweeds [11]. Fx has shown a potential ability to lower lipid peroxidation [12] and exert anti-inflammatory [13], anti-tumor [14], and anti-hyperuricemia [15] effects. Additionally, in cardiovascular disease Fx has shown recovery effects related to DNA damage and cardioprotective effects [16]. However, studies of the protective role of Fx in heart valve interstitial cells are lacking. In this study, we investigated the potential mechanism of Fx in protecting the heart valves from fibrosis.

## 2. Results

### 2.1. Protective Effect of Fx on VIC Cell Viability from H_2_O_2_-Induced Oxidative Stress

#### 2.1.1. Effect of H_2_O_2_ on the VIC Cell Viability

**Figure 1:**
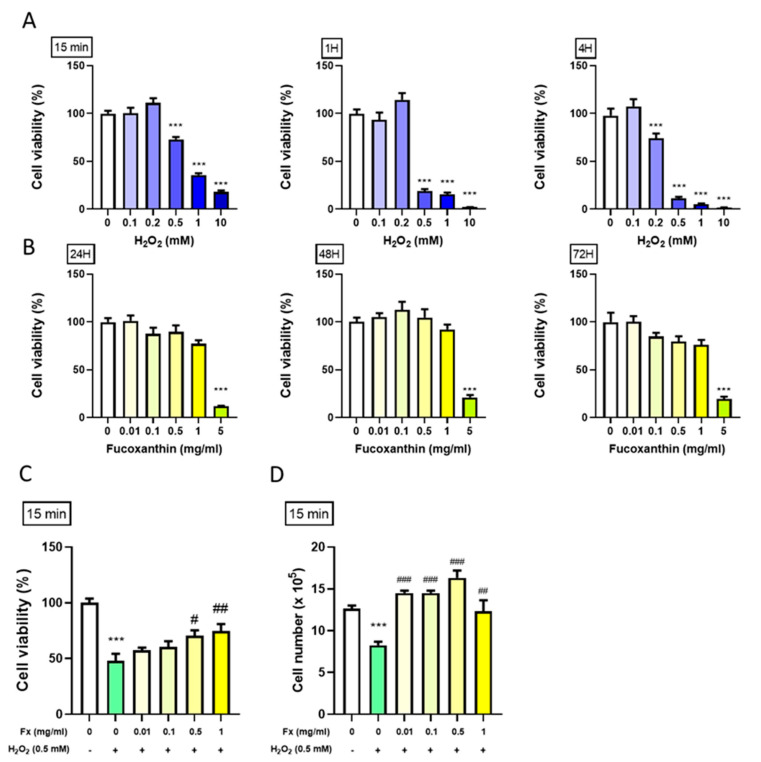
Protective effect of fucoxanthin (Fx) on VIC cell viability following H_2_O_2_-induced oxidative stress. Rat heart valve interstitial cells (3000 cells/well) were cultured in DMEM/F12 supplemented with 10% FBS for 24 h. (**A**) Treated with H_2_O_2_ (0.1–10 mM) for 15 min, 1 h and 4 h. (**B**) Treated with Fx (0.01–5 mg/mL) for 24, 48, and 72 h. (**C**, **D**) Cells were pretreated with Fx (Fx, 0.01–1 mg/mL) for 24 h, then treated with 0.5 mM of H_2_O for 15 min. Cell viability was analyzed by MTT assay in (**C**) or through cell counting in (**D**). ***, *p* < 0.001 compared with untreated control cells. ^#^, *p* < 0.05; ^##^, *p* < 0.01; ^###^, *p* < 0.001 compared with the H_2_O_2_-induced group. White bar, control group; Blue bar, different dosage of H_2_O_2_-induced group; Green bar, H_2_O_2_-induced group; Yellow bar, Fx-treated group.

After the cell extraction (Supplementary Figure S1), we treated the cells with H_2_O_2_ in serial doses for 15 min, 1 h and 4 h to induce high oxidative stress in VIC to measure the cell viability. The results showed that H_2_O_2_ at doses of 0.5 mM and above significantly reduced cell proliferation at all the time points. Notably, H_2_O_2_ at 0.5 mM for 15 min (min) decreased the cell viability by 30%, which is considered a moderate effect of oxidative stress to be used in following experiments (Figure 1A).

#### 2.1.2. Effect of Fx on VIC Cell Viability

To evaluate Fx’s effect on the VIC cell viability, we treated the cells with different doses of Fx for 24, 48, and 72 h and measured cell viability using and 3-(4,5-Dimethylthiazol-2-yl)-2,5-Diphenyltetrazolium Bromide (MTT) assay. The results showed that Fx did not significantly affect the VIC cell viability, except when the highest dosage (5 mg/mL) was used, probably due to its toxic effect (Figure 1B).

#### 2.1.3. Fx-Abrogated H_2_O_2_-Induced VIC Viability Change

Next, we explored the combined effect of the Fx and H_2_O_2_ on VIC viability to evaluate the protective effect of Fx. Cells were first pretreated with different doses of Fx for 24 h, then exposed to 0.5 mM of H_2_O_2_ for 15 min., which earlier was enough to inhibit cell proliferation. MTT assay was used to measure the cell viability. The results showed that Fx could alleviate the inhibitory effect of H2O2-induced oxidative stress on VIC growth (Figure 1C,D).

### 2.2. Fx-Ameliorated H_2_O_2_-Induced DNA Damage and Apoptosis-Related Protein Expression

#### 2.2.1. Fx Decreased H_2_O_2_-Induced VIC Cell Morphology Changes and DNA Damage

**Figure 2:**
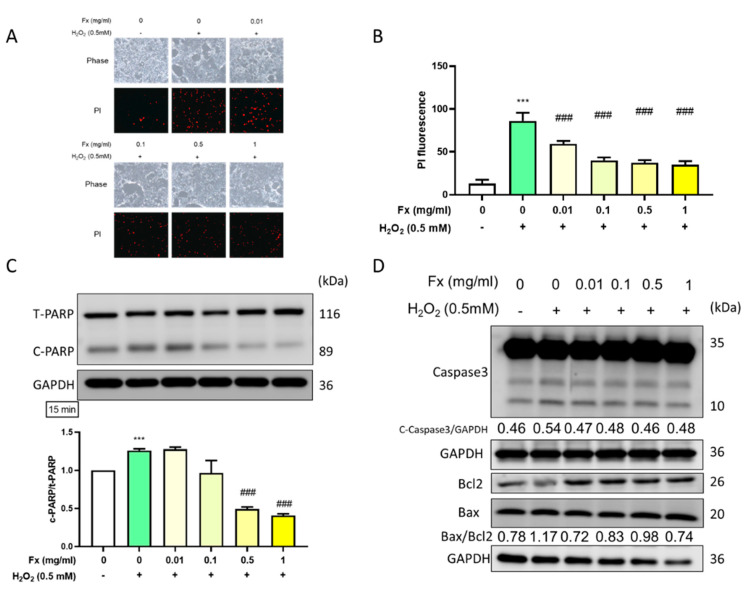
Fucoxanthin (Fx) ameliorated H_2_O_2_-induced DNA damage and apoptosis-related protein expression. Rat heart valve interstitial cells were cultured in a 6-well plate for 24 h, pretreated with Fx for 24 h, and then treated with H_2_O_2_ for 15 min. (**A**) Cells were stained with PI solution (1 μg/mL) for 1 h and visualized by microscopy at 200× magnification. (**B**) Quantified by Image J. Western blotting was used to explore the protein expression of (**C**) total and cleaved PARP, and (**D**) apoptosis-related markers cleaved caspase 3, Bcl_2_, and Bax. ***, *p* < 0.001 compared with untreated control group. ^###^, *p* < 0.001 compared with H_2_O_2_-induced group. White bar, control group; Green bar, H_2_O_2_-induced group; Yellow bar, Fx-treated group.

To further explore the protective effect of Fx on VIC cells in the context of halting DNA damage, we used PI staining and microscopy to visually observe any morphological changes. Following the pretreatment of the cells with Fx and then H_2_O_2_, PI staining with fluorescence signal increased, the result showed that Fx was able to prevent oxidative stress-induced DNA damage (Figure 2A,B).

#### 2.2.2. Fx-Ameliorated H_2_O_2_-Induced Apoptosis-Related Protein Expression

Along with the increase in cell damage and decrease in cell viability, we used the Western blotting technique to explore the effect of the administration of both H_2_O_2_ and Fx on the apoptosis-related protein expression. The H_2_O_2_-induced group showed a significant increase in the expression of the cleaved form of PARP, while pretreatment with Fx significantly reversed the expression of cleaved PARP (Figure 2C). Furthermore, we explored other apoptosis-related markers, such as cleaved caspase 3 and the Bax/Bcl2 ratio (Figure 2D). The results confirmed that H_2_O_2_-induced apoptosis, which was reversed after the Fx treatments. Collectively, these results highlight the anti-apoptotic effect of Fx in VIC cells.

### 2.3. Effect of Fx on H_2_O_2_-Induced Reactive Oxygen Species

**Figure 3:**
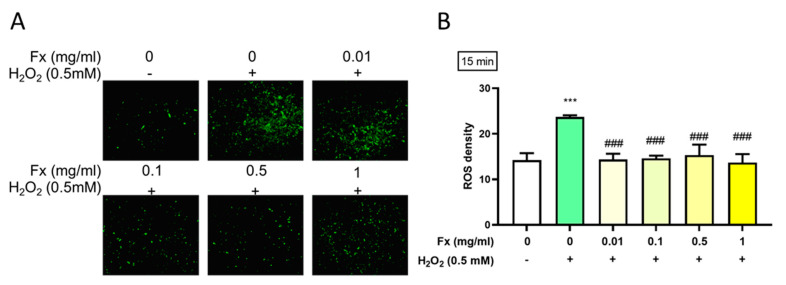
Effect of fucoxanthin (Fx) on the H_2_O_2_-induced ROS level. Rat heart valve interstitial cells were cultured in DMEM/F12 supplemented with 10% FBS for 24 h, pretreated with 0.01–1 mg/mL of Fx for 24 h, then treated with 0.5 mM of H_2_O_2_ for 15 min. ROS were visualized using DCFDA. These were assessed by microscopy at 200× magnification (**A**) and we used Image J for the density quantification (**B**). ***, *p* < 0.05 compared with untreated control group; ^###^, *p* < 0.001 compared with H_2_O_2_ group. White bar, control group; Green bar, H_2_O_2_-induced group; Yellow bar, Fx-treated group.

According to DCFDA’s result, treatment with H_2_O_2_ induced a high level of ROS, which in turn could lead to damage to the valve structure and subsequent calcification [17]. The results show that Fx could alleviate the high oxidative stress-induced ROS level, as shown by a reduction in the fluorescence (Figure 3A) and density (Figure 3B).

### 2.4. Effect of Fx on Oxidative Stress-Induced Calcification and the Expression of Its Related Markers in VIC

**Figure 4:**
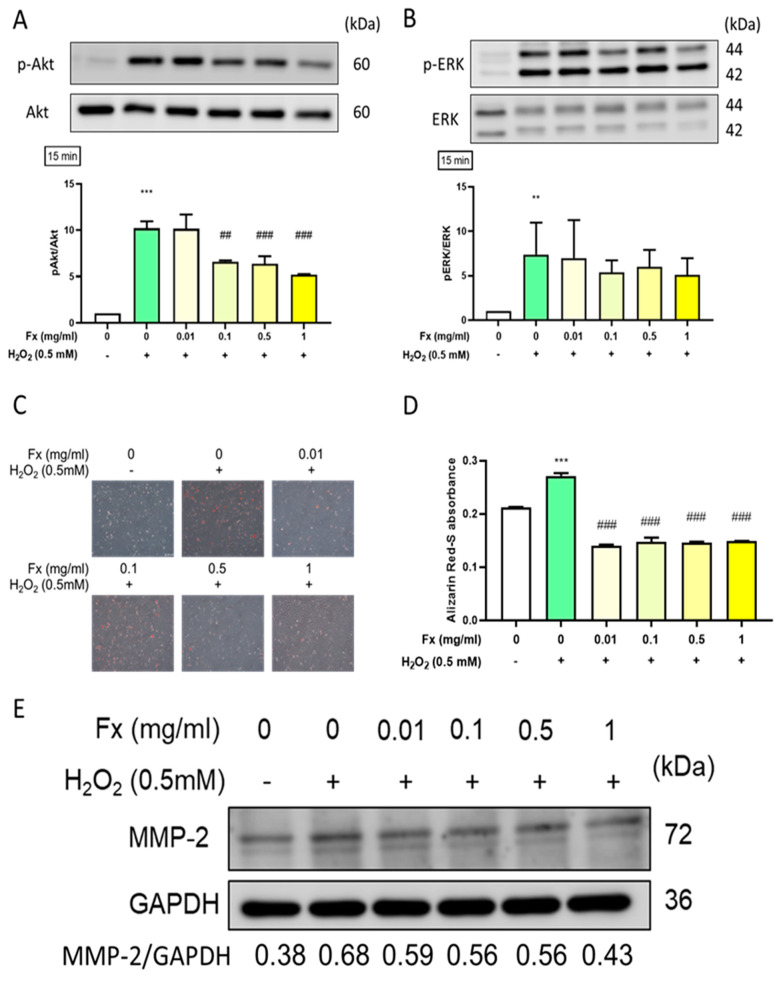
Effect of fucoxanthin (Fx) on oxidative stress-induced calcification and the expression of its related markers in VIC. Rat heart valve interstitial cells were cultured in a 6-well plate for 24 h, pretreated with Fx for 24 h, then induced with H_2_O_2_ for 15 min. Western blotting was used to analyze the protein expression of (**A**) pAkt/Akt (**B**) pERK/ERK. (**C**) Alizarin Red-S staining was used to visualize calcification, which was assessed by microscopy at a 200× magnification and (**D**) quantified by Image J. (**E**) MMP-2 protein expression was analyzed using Western blotting. **, *p* < 0.01; ***, *p* < 0.001 compared with untreated control group. ^##^, *p* < 0.01; ^###^, *p* < 0.001 compared with H_2_O_2_-induced group. White bar, control group; Green bar, H_2_O_2_-induced group; Yellow bar, Fx-treated group.

Oxidative stress was shown to trigger calcification in VIC [18], which involves the activation of the Akt/ERK signaling pathway [19]. Therefore, we evaluated the effect of both H_2_O_2_ and Fx on the ROS and calcification-related Akt/ERK signaling pathway. The results showed that H_2_O_2_ treatment significantly increased the phosphorylation of Akt and ERK proteins. Notably, the pre-treatment with Fx was able to decrease such activation (Figure 4A,B). Furthermore, we used Alizarin Red-S for calcification staining and the results showed that calcification was increased in response to H_2_O_2_ and alleviated by Fx pretreatment (Figure 4C,D). Consistently, the Western blotting results showed that the Fx treatment was able to partially oppose the H_2_O_2_-induced ECM remodeling of marker matrix metalloproteinase 2 (MMP-2) in VIC cells (Figure 4E).

### 2.5. Long-Term Cardioprotective Effect of Fx Treatment in Dogs

**Figure 5:**
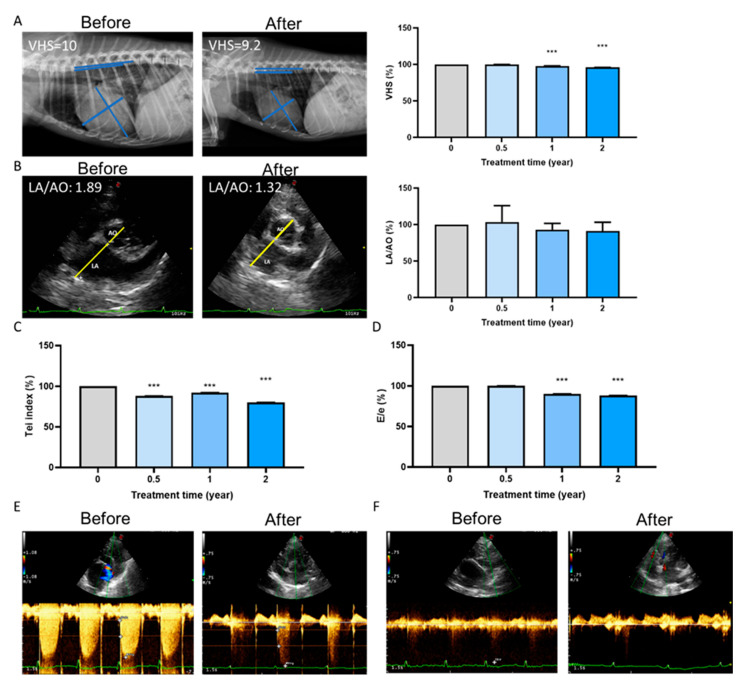
Long-term cardioprotective effect of fucoxanthin (Fx) treatment in dog. After Fx treatments, echocardiography analysis showed (**A**) a significant decrease in the VHS score, (**B**) an improvement in LA/AO, (**C**) a reduction in the Tei index, and (**D**) a decrease in the E/e percentage. The linkage of (**E**) the mitral valve and (**F**) the tricuspid valve decreased. ***, *p* < 0.001 compared with the baseline.

Long-term treatment with Fx in the 26 dogs resulted in a significant decrease in their vertebral heart size (VHS) (Figure 5A). VHS score is a number that normalizes heart size to body size using the mid-thoracic vertebrae as units of measurement, reflecting compensatory cardiac enlargement [20]. In addition, treatment showed the improvement of the left atrium to aortic (LA/AO) dimension ratio (Figure 5B), Tei index (Figure 5C), and E/e value (Figure 5D). The linkage of the mitral valve and tricuspid valve showed a significant decrease in echocardiography (Figure 5E,F). Collectively, Fx supplementation could improve the overall function of ventricular contraction and relaxation, confirming the findings of previous studies [21].

## 3. Discussion

Oxidative stress and ROS contribute significantly to the pathogenesis of cardiovascular diseases [22], such as stroke and hypertension [23]. Elevated ROS could cause vascular damage via recruiting more leukocytes in blood reperfusion [24]. Moreover, oxidative stress can induce endothelial dysfunction and induce pro-fibrotic and pro-osteoblastic effects with subsequent calcification in the aortic valves, as shown in a mouse calcific aortic valve disease (CAVD) model [25].

Oxidative stress via ROS induces damage to DNA double strands, in addition to the rapid phosphorylation of H2AX by PI3K-related kinases with downstream Akt modulation [26]. Additionally, Akt phosphorylation induced the osteogenic early marker RUNX2 [17], which had the ability to increase osteogenic differentiation [27]. The progression of calcification in VIC cells also involved a significant increase in the osteogenic medium (OM) condition as well as the increased phosphorylation of the NF-κB, PI3K-Akt, TNF, and MAPK signaling pathways [19]. In this study, Fx treatment could alleviate the oxidative stress-induced ROS level (Figure 3) and decrease the progression calcification through Akt- and MAPK-related signaling pathways (Figure 4).

Aortic leaflets are composed of three main layers, named the fibrosa, spongiosa, and ventricularis. This composition builds up the valve function, which is mainly diastole and systole extension [28]. Considering that ECM is also the main component of valves, the structure and the arrangement of ECM play important roles in the valve function.

Increased inflammation and LDL oxidation induce the calcification of the valves [29]. On the other hand, oxidized high-density lipoprotein (ox-HDL) increases in CAVD patients’ plasma to protect against the high oxidative stress induced by CAVD [30]. As ROS increase, chronic inflammation, with accompanying immunity components, increases, along with increased growth factors and proteolytic enzymes, such as MMP-9 and MMP-2 for ECM deposition and remodeling effects [31]. Therefore, the up-regulation of MMPs indicates valve remodeling and calcification [32].

Several studies have shown vascular diseases with ROS-related pathology in studies of animals fed with a high-cholesterol or high-fat diet to induce vascular calcification. There are different categories of antioxidant compound. Natural antioxidants, such as gallic acid [33], curcumin [34], and quercetin [35], or synthetic ROS scavengers such as N-acetylcysteine, pyrrolidine dithiocarbamate, and poly(1,8-octamethylene-citrate-co-cysteine) with a reduction in ROS, PI3K/Akt or inflammation-related protein expression could reduce calcification and apoptosis through ROS scavenging [36].

Fx exerts antioxidant ability that has been shown to eliminate pregnancy-related hypertension [37], high glucose-induced diabetes retinopathy [38], and ox-LDL-induced endothelial damage [39] via ROS reduction. In our current in vitro study, Fx showed a potential protective effect against high oxidative stress-induced VIC damage through a reduction in apoptosis (Figure 2) and ROS and modulation of the phosphorylation of Akt and ERK to decrease the calcification and ECM accumulation (Figure 4).

The first-line diagnosis of heart valve disease in dogs includes the use of radiographs, ultrasound, and echocardiography [40]. Indicators of valve disease include an increase in VHS score or LA/AO. In echocardiography assessment, the veterinarian would use the Tei index to evaluate systolic and diastolic function. The E/e’ ratio is used to assess the mitral valve inflow and analyze the mitral valve leakage and left ventricular diastolic dysfunction [41]. In our study, fucoxanthin treatments could reverse mitral valve and tricuspid valve leakage. Moreover, using in vivo experiments in dogs, we showed that the long-term supplementation of Fx could improve both compensatory cardiac hypertrophy and valve function (Figure 5).

## 4. Materials and Methods

### 4.1. Reagents Preparation

High-stability fucoxanthin (HS Fucoxanthin, HSFUCO, Fx) was obtained from Hi-Q Marine Biotech International Ltd. (Taipei, Taiwan) and dissolved in ddH_2_O [38].

### 4.2. Cell Extraction and Treatment

The extraction of primary rat valve interstitial cells (VIC) was carried out as shown in a previous study [42]. Briefly, after harvesting all the leaflets, pellet was centrifuged and incubated with collagenase II (Thermo) for 2 h to obtain VIC debris. Cells were then cultured in Dulbecco’s Modified Eagle’s Medium (DMEM/F12) (CASSION, Taichung City, Taiwan) combined with 100 units/mL of penicillin, 100 μg/mL of streptomycin (CORNING, Manassas, VA, USA), sodium bicarbonate (2.438 g/L; Bio-Shop, Burlington, ON, Canada), and 10% fetal bovine serum (FBS; CORNING, Manassas, VA, USA). To explore the protective effect of Fx against oxidative stress, VIC were pretreated with Fx for 24 h and then treated with H_2_O_2_ for 15 min.

### 4.3. 3-(4,5-Dimethylthiazol-2-yl)-2,5-Diphenyltetrazolium Bromide (MTT) Assay

VIC cells were seeded in 96-well plates (3000 cells/well). The cells were pretreated with different doses of Fx (mg/mL) for 24 h and then with H_2_O_2_ for 15 min. After the treatment, we used the MTT assay (Abcam, Cambridge, MA, USA) for the analysis of cell viability. We added 1 mg/mL of MTT for 3 h until the crystal precipitation formed. Then added 100 μL/well of dimethyl sulfoxide (DMSO; ECHO Chemical Co. Ltd., Taipei, Taiwan) to dissolve the crystal formation. We used VERSA Max microplate reader (Molecular Devices, San Jose, CA, USA) to measure the optical density at 570 and 630 nm.

### 4.4. Cell Counting

After the treatments, cell pellets were mixed with 0.4% trypan blue solution (Gibco, Grand Island, NY, USA). We used a hemocytometer (Hausser scientific company, Horsham, PA, USA) to calculate the cell number at 200× magnification.

### 4.5. PI Staining

Propidium iodide (PI) solution (500 μg/mL) (Sigma-Aldrich, St. Louis, MO, USA) was dissolved with sterile ddH_2_O and stained with PI (1 μg/mL) solution for 1 h. We used microscopy (Olympus, Tokyo, Japan) to carry out fluorescence imaging at 200× magnification.

### 4.6. ROS Density Measurement

VIC cells were cultured in 6-well plates. We used 25 μM 2′,7′–dichlorofluorescin di-acetate (DCFDA, Cayman, Ann Arbor, MI, USA) staining for 30 min. Then, we used microscopy to capture the fluorescence image. We used the Image J software (Version 1.52t, NIH, Bethesda, MD, USA) to carry out ROS density quantification in single cells.

### 4.7. Alizarin Red-S Staining Assay

The calcification progression was assessed by Alizarin Red-S staining (Sciencell, Carlsbad, CA, USA). After treatment, the cells were fixed with 4% paraformaldehyde (Sigma-Aldrich) for 10 min and then stained with Alizarin Red-S for 30 min. We used a fluorescence microscope to image the stained area. We used the VERSA Max microplate reader to measure the absorbance at 405 nm.

### 4.8. Protein Extraction and Western Blot

Cells were lysed in radioimmunoprecipitation assay (RIPA) lysis buffer with added protease and phosphatase inhibitors (Roche, Mannheim, Baden-Württemberg, Germany). Additionally, we quantified the cells with a bicinchoninic acid (BCA) assay, used sodium dodecyl sulfate polyacrylamide gel electrophoresis (SDS-PAGE), and transferred to a polyvinylidene fluoride (PVDF) membrane. Blocking with 5% bovine serum albumin (BSA) solution for 1 H was carried out. We used primary antibodies—poly (ADP-ribose) polymerase (PARP) (1:1000; Cell Signaling, Boston, MA, USA), glyceraldehyde 3-phosphate dehydrogenase (GAPDH) (1:10,000; Proteintech, Rosemont, IL, USA), p-Akt (1:1000, Cell signaling), Akt (1:1000, Cell signaling), p44/42 MAPK (Erk1/2) (1:1000, Cell signaling), phospho-p44/42 MAPK (Erk1/2) (1:1000, Cell signaling), MMP-2 (1:1000, Abcam), BCL2 Associated X (Bax) (1:1000, Cell signaling), and B-cell lymphoma 2 (Bcl-2) (1:500, Santa Cruz, Santa Cruz, CA, USA)—cultured the mixture at 4 °C overnight, washed it three times, and then stained it with horseradish peroxidase (HRP)-conjugated secondary antibody (1:5000–10,000) for 2 h. The signal was captured by the eBlot Touch Imager^tm^ (eBlot Photoelectric Technology, Shanghai, China). The band densities were determined using the Image J software program version 1.52 t (NIH, Bethesda, MD, USA). The expression level of these target proteins was analyzed in three individual experiments.

### 4.9. In Vivo Animal Experiments

With the help of a veterinarian, we recruited 26 heart disease-diagnosed dogs for the in vivo experiment. The dogs were treated with Fuco Pets HeartFight^®^ (contained 60 mg/kg Fx) twice daily from Hi-Q Marine Biotech International Ltd. (Taipei, Taiwan) combined with medical treatments for 0.5 to 2 years. We used conventional echocardiography and standard Doppler examination to follow up the valve function. Esaote’s MyLab™ClassC^®^ (Italy) equipped with a PA-122 probe cardio phased array (frequency range of 3–8 MHz) was used to obtain all the echocardiographic data.

The left atrium to aorta (LA/AO) ratio was measured using B-mode images acquired from a short axis five-chamber view of the right sternum wall.

### 4.10. Statistical Analysis

The data are expressed as the mean ± standard deviation (SD). We used GraphPad Prism 8.0 for the analysis. Student’s t-test was used for the comparisons between the two groups. One-way ANOVA tests were used to compare multiple groups, followed by Tukey’s post hoc test. A *p*-value of less than 0.05 was considered significant. The p values are presented as *, *p* < 0.05; **, *p* < 0.01; ***, *p* < 0.001; or ^#^, *p* < 0.05; ^##^, *p* < 0.01; and ^###^, *p* < 0.001.

## 5. Conclusions

**Figure 6:**
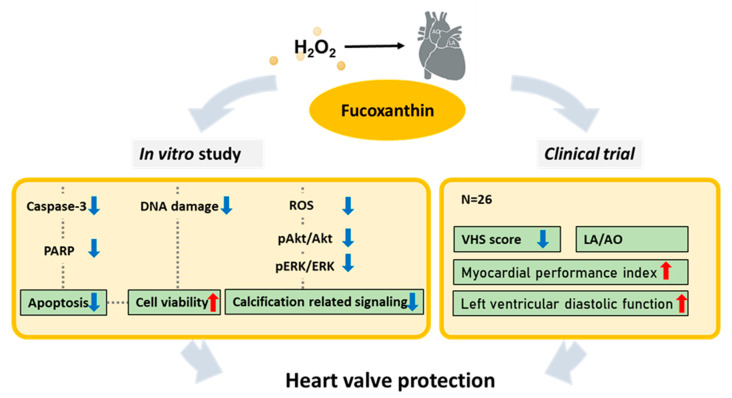
Schematic representation of the potential effects of Fx on protection against high oxidative stress-related cell apoptosis and the ROS-related calcification signaling pathway. Furthermore, in vivo experiment shows Fx’s ability to protect against valve-related disease in dogs.

Treatment with Fx was demonstrated to effectively protect against the harmful effects of high H_2_O_2_-induced oxidative stress in heart valve interstitial cells through the antioxidant potential of Fx as follows: (1) Fx can recover H_2_O_2_-induced cell viability impairment, (2) Fx can oppose H_2_O_2_-induced apoptosis, (3) Fx can inhibit the Akt/ERK-related signaling pathway to reduce heart valve calcification, (4) long-term treatment with Fx could recover the heart valve function and leakage in dogs. These data show that Fx has the potential to protect heart valve cells from damage caused by high oxidative stress (Figure 6).

## Data Availability

The data presented in this study are available on request from the corresponding author.

## Supplementary Materials

The following are available online at https://www.mdpi.com/article/10.3390/md19060307/s1: Figure S1. Protein marker expression in rat valve interstitial cells (A) Used western blot Rat valve interstitial cells (VICs) were negative for the endothelial cell marker, CD31, positive with vimentin and α-SMA. (B) Used microscopy in 200× magnification to capture the morphology of VICs.
