# Supplementary material for: Protective Effects of Fucoxanthin on Hydrogen Peroxide-Induced Calcification of Heart Valve Interstitial Cells
Source: Mar Drugs. 2021 May 26;19(6):307. doi: 10.3390/md19060307 (PMC8227531; doi:10.3390/md19060307)
Supplement: Supplementary file 1 [file marinedrugs-19-00307-s001.zip › marinedrugs-1213964-supplementary.pdf]

Protective effects of fucoxanthin on hydrogen peroxide-induced calcification of heart valve interstitial cells

Yi-Fen Chiang [1], Chih-Hung Tsai [2]†, Hsin-Yuan Chen [1,3]†, Kai-Lee Wang [4], Hsin-Yi Chang[5], Yun-Ju Huang [1], Yong-Han Hong [3], Mohamed Ali [6], Tzong-Ming Shieh [7], Tsui-Chin Huang [8] Ching-I Lin [9], Shih-Min Hsia [1,5,10,11,*]

[1] School of Nutrition and Health Sciences, College of Nutrition, Taipei Medical University, Taipei 11031, Taiwan; yvonne840828@gmail.com (Y.-F.C.); hsin246@gmail.com (H.-Y.C.); d04641004@ntu.edu.tw (Y.-J.H.);

[2] Yu-Kang Animal Hospital, New Taipei City, Taiwan; yukangdvm@yahoo.com.tw (C.-H.T.)

[3] Department of Nutrition, I-Shou University, Kaohsiung 84001, Taiwan; hsin246@gmail.com (H.-Y.C.); yonghan@isu.edu.tw (Y.-H. H)

[4] Department of Nursing, Ching Kuo Institute of Management and Health, Keelung 20301, Taiwan; kellywang@tmu.edu.tw (K.-L.W.)

[5] Graduate Institute of Metabolism and Obesity Sciences, Taipei Medical University, Taipei, Taiwan; hsinyi.chang@tmu.edu.tw (H.-Y.C.)

[6] Clinical Pharmacy Department, Faculty of Pharmacy, Ain Shams University, 11566 Cairo, Egypt; mohamed.aboouf@pharma.asu.edu.eg (M.A.)

[7] School of Dentistry, College of Dentistry, China Medical University, Taichung 40402, Taiwan; tmshieh@mail.cmu.edu.tw (T.-M.S.)

[8] Graduate Institute of Cancer Biology and Drug Discovery, College of Medical Science and Technology, Taipei Medical University, Taipei 11031, Taiwan; tsuichin@tmu.edu.tw (T.-C.H.)

[9] Department of Nutrition and Health Sciences, Kainan University, Taoyuan 338, Taiwan. cilin@mail.knu.edu.tw (C.-I.L.)

[10] School of Food and Safety, Taipei Medical University, Taipei 11031, Taiwan

[11] Nutrition Research Center, Taipei Medical University Hospital, Taipei 11031, Taiwan

* Correspondence: bryanhsia@tmu.edu.tw (S.-M.H.); Tel.: +886-273-61661-6558

† Equal contribution.

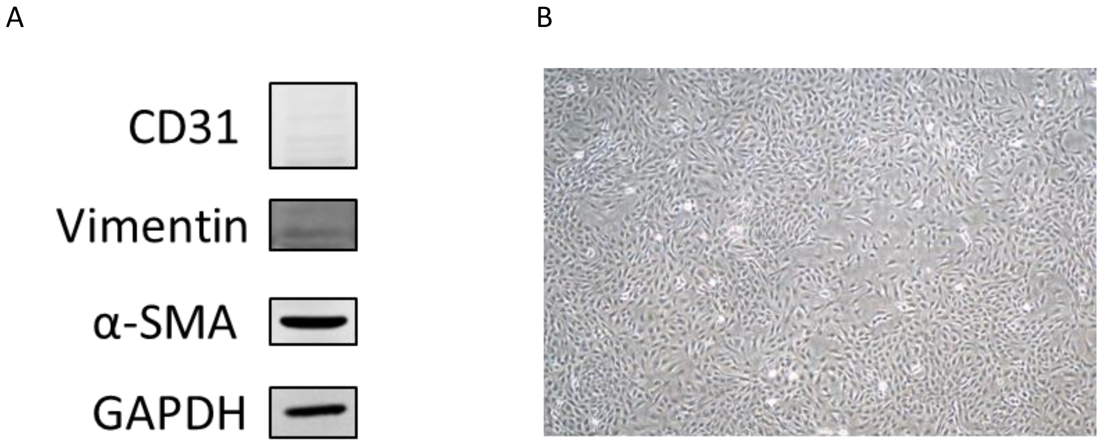

Figure S1. Protein marker expression in Rat valve interstitial cells. (A) Used western blot Rat valve interstitial cells (VICs) were negative for the endothelial cell marker, CD31, positive with vimentin and α-SMA. (B) Used microscopy in 200X magnification to capture the morphology of VICs
